# Factors Associated with Lip-Closing Strength in Children Undergoing Orthodontic Assessment: A Cross-Sectional Study

**DOI:** 10.3390/diagnostics16183065

**Published:** 2026-09-21

**Authors:** Linxian Fang, Jin Zhao, Jian Wang, Dingzhuo Jiang, Tao Qiu, Ying Shi, Yafen Zhu, Zhifang Wu

**Affiliations:** 1 Stomatology Hospital, School of Stomatology, Zhejiang University School of Medicine, Hangzhou 310000, China; 2 Zhejiang Provincial Clinical Research Center for Oral Diseases, Zhejiang Key Laboratory of Oral Biomedical, Hangzhou 310000, China

**Keywords:** lip-closing strength, skeletal malocclusion, children, mouth-breathing screening, orthodontic assessment

## Abstract

**Background/Objectives:** Lip-closing strength (LCS) is an objective measure of perioral muscle function, yet its associations with sagittal and vertical skeletal patterns and mouth-breathing screening status in school-aged children have not been well characterized. This study aimed to investigate the associations between LCS and demographic characteristics, sagittal and vertical skeletal patterns, and mouth-breathing screening status in children undergoing orthodontic assessment. **Methods:** A cross-sectional study was conducted on 379 children aged 7–12 years presenting for initial orthodontic evaluation. LCS was measured using a custom-built dynamometer with a lip-button attachment. Sagittal and vertical patterns were classified by lateral cephalometry (ANB, SN–MP angles), and mouth-breathing screening status was assessed using a validated questionnaire (Habitual Mouth-Breathing Score ≥ 4 defined as screen-positive). Multivariable linear regression was performed with LCS as the outcome. **Results:** The mean LCS was 4.61 ± 1.58 N. Skeletal Class II (4.41 ± 1.33 N) and screen-positive mouth breathing (median 3.97 N) were associated with significantly lower LCS compared to Class I and screen-negative participants, respectively. Age, sex, and vertical pattern showed no significant association. In adjusted analysis, skeletal Class I (B = 0.575, *β* = 0.180, 95% CI: 0.268–0.882) and Class III (B = 0.867, *β* = 0.188, 95% CI: 0.416–1.317) were associated with significantly greater LCS relative to Class II (*p* < 0.001 for both), whereas screen-positive mouth breathing was associated with significantly lower LCS (B = −0.808, *β* = −0.267, 95% CI: −1.099 to −0.517, *p* < 0.001). **Conclusions:** Lower LCS was associated with skeletal Class II pattern and positive mouth-breathing screening status. The observed differences were modest, and their clinical significance remains uncertain. LCS should not be used as a standalone diagnostic or treatment-planning indicator.

## 1. Introduction

Lip-closing strength (LCS) refers to the maximal force produced by the orbicularis oris muscle complex. It provides an objective measure of perioral muscle function. Adequate LCS is important for maintaining normal lip closure and supporting dentofacial soft tissue balance. Reduced LCS has been associated with altered dentofacial soft tissue balance and malocclusion [1]. The relationship between lip strength and malocclusion was quantified as early as 1986 by Thüer and Ingervall using a dynamometer [2]. Previous studies have shown that LCS increases during early childhood [3]. Lip and tongue pressures also continue to increase during childhood and adolescence [4]. LCS usually reaches a stable level by school age [3].

In addition to normal growth, pathological conditions may also influence lip function. Chronic mouth breathing is considered an important factor associated with altered perioral muscle function. This condition changes the resting position of the lips and decreases perioral muscle activity. These alterations are commonly associated with Class II skeletal patterns and an open-lip “adenoid” facial appearance [5].

Previous epidemiological studies have consistently reported a higher prevalence of malocclusion among children with mouth-breathing habits [6]. In preschool children, mouth-breathing syndrome has been related to incompetent lip seal [7], and inadequate lip closure has also been identified as an independent factor associated with malocclusion [8]. Large population-based studies have shown that up to 30.7% of children present with inadequate lip closure [9]. This condition is particularly frequent among children with skeletal Class II malocclusion [10].

Although previous pediatric studies have described developmental changes in LCS and oral muscle pressures [3,4,11], evidence directly relating LCS to craniofacial skeletal patterns and mouth-breathing screening status in school-aged children remains limited. Sex-related differences in LCS were not observed in preschool children [11], whereas such differences have been reported in adults [12]. Research in an East Asian pediatric population has suggested a relationship between skeletal classification and orofacial muscle function, although tongue pressure rather than LCS was evaluated [13]. Therefore, whether LCS differs according to sagittal and vertical skeletal patterns and mouth-breathing screening status during childhood remains unclear.

To address these gaps, this cross-sectional study evaluated 379 children aged 7–12 years who underwent orthodontic assessments. The study investigated factors associated with LCS, including age, sex, sagittal and vertical skeletal patterns, and mouth breathing. It also explored the potential role of LCS as an adjunctive measure of perioral muscle function during orthodontic evaluation.

## 2. Materials and Methods

### 2.1. Study Design and Reporting

This cross-sectional study was designed and reported according to the Strengthening the Reporting of Observational Studies in Epidemiology (STROBE) guidelines [14]. The study was conducted in accordance with the Declaration of Helsinki and was approved by the Medical Ethics Committee of the Stomatology Hospital, Zhejiang University School of Medicine, China. Written informed consent was obtained from the guardians of all participants before enrollment.

### 2.2. Study Population and Setting

The study population comprised children in the mixed dentition and early permanent dentition stages who were referred for orthodontic assessment. Participants were consecutively recruited from the Department of Pediatric Dentistry at the Stomatology Hospital, Zhejiang University School of Medicine, China, between November 2024 and November 2025.

The inclusion criteria were as follows: (1) age 7–12 years; (2) ability to cooperate during LCS measurement; and (3) availability of complete clinical and imaging records. The exclusion criteria were as follows: (1) previous orthodontic or orthognathic treatment; (2) severe oral and maxillofacial diseases or conditions that could substantially affect lip function, craniofacial morphology, or LCS measurement, including previous maxillofacial trauma, maxillofacial tumors or cysts, previous maxillofacial surgery, severe oral mucosal diseases, and neuromuscular disorders affecting the orofacial region; (3) pre-existing structural or functional lip dysfunction that could affect lip closure or perioral muscle function, including residual lip dysfunction after cleft lip repair and other clinically evident lip dysfunction; and (4) temporomandibular disorders (TMD). A history of language or speech therapy was not systematically collected and was therefore not used as an exclusion criterion. TMD-related history was assessed through routine medical and dental history taking at the initial visit. Routine clinical examination included assessment of mouth opening, joint clicking, and mandibular deviation during mouth opening. This assessment was intended to identify clinical findings suggestive of TMD rather than to classify specific TMD subtypes. Patients with a known history of TMD or clinical findings suggestive of TMD were advised to undergo further evaluation by an oral and maxillofacial surgeon and were excluded if TMD was confirmed. A dedicated TMD diagnostic questionnaire or formal TMD classification system was not used in the present study.

**Figure 1:**
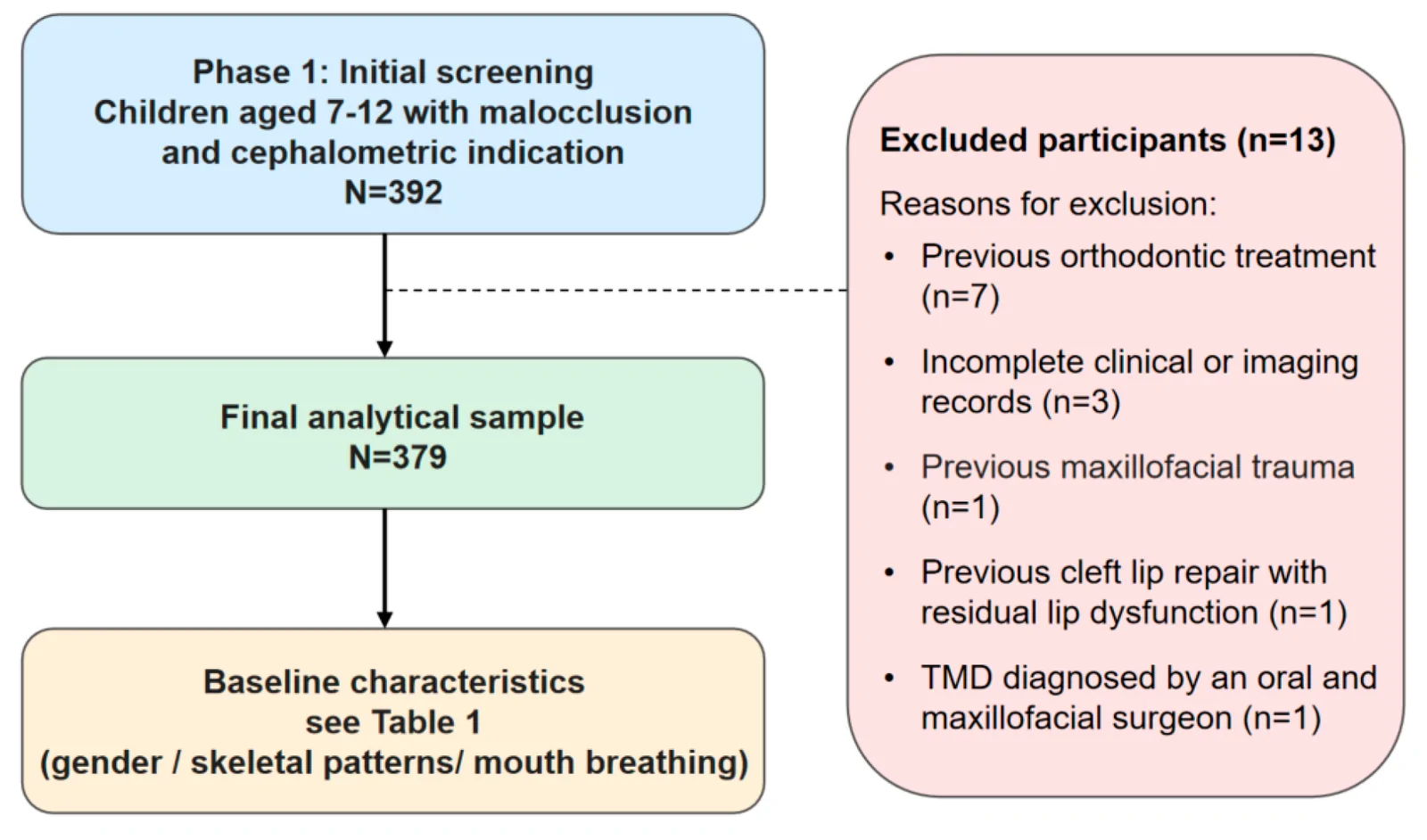
STROBE flow diagram of participant recruitment and exclusion. Baseline characteristics see Table 1.

During the recruitment period, 392 children were consecutively screened. Thirteen children were excluded due to previous orthodontic treatment (*n* = 7), incomplete clinical or imaging records (*n* = 3), previous maxillofacial trauma (*n* = 1), previous cleft lip repair with residual lip dysfunction (*n* = 1), or a documented diagnosis of TMD by an oral and maxillofacial surgeon in the medical record, with joint clicking noted on routine clinical examination (*n* = 1). The remaining 379 children were included in the final analysis. The participant recruitment and exclusion process is presented in a STROBE flow diagram (Figure 1).

**Table 1:** Baseline demographic and clinical characteristics of the study participants (*N* = 379).

	Characteristic Category	*n* (%) or Mean ± SD
Sex	Male	194 (51.2)
	Female	185 (48.8)
Age (years)	-	8.96 ± 1.50
	7	70 (18.5)
	8	104 (27.4)
	9	72 (19.0)
	10	59 (15.6)
	11	51 (13.5)
	12	23 (6.1)
Sagittal skeletal pattern	Class I	95 (25.1)
	Class II	246 (64.9)
	Class III	38 (10.0)
Vertical skeletal pattern	Low-angle type	44 (11.6)
	Normal-angle type	189 (49.9)
	High-angle type	146 (38.5)
Mouth breathing	Screen-positive	113 (29.8)
	Screen-negative	266 (70.2)

### 2.3. Sample Size

An a priori sample size calculation was performed using G*Power (version 3.1.9.7; Heinrich-Heine-Universität Düsseldorf, Düsseldorf, Germany). Based on previous evidence suggesting an association between reduced perioral muscle function and skeletal Class II characteristics, the primary comparison was predefined as the difference in LCS between skeletal Class II and non-Class II participants. A two-sided independent-samples *t*-test was specified with α = 0.05 and 80% statistical power.

Preliminary data from the first 30 consecutively enrolled children aged 7–12 years in our department were used to estimate the variability of LCS. The mean LCS was 4.6 N, with a standard deviation of 1.5 N. The pooled standard deviation was therefore estimated as approximately 1.5 N. A mean difference of 0.5 N between groups was assumed for sample size estimation, corresponding to Cohen’s d ≈ 0.33. The calculation indicated that 142 participants were required in each group. Considering a 10% non-evaluable rate, the target sample size was set at 315 participants.

The final sample included 379 children, exceeding the calculated requirement and providing adequate statistical power for the primary Class II versus non-Class II comparison.

### 2.4. Data Collection

#### 2.4.1. Demographic and Clinical Data

Demographic information, including age and sex, was extracted from clinical records. Age and sex were selected a priori as basic demographic variables because previous studies have reported developmental and sex-related differences in lip-closing function across different age groups [3,11,12,15]. The assessment of mouth-breathing tendency was conducted using the validated 10-item Habitual Mouth-breathing (HMB) questionnaire, which was completed by the parents [16]. Each item on the questionnaire was rated on a 3-point scale (0–2), resulting in a total possible score ranging from 0 to 20. Scores between 0 and 3 indicate predominant nasal breathing, whereas scores of 4 or higher suggest suspected or established habitual mouth breathing [16]. Given the high prevalence of mouth breathing among children seeking orthodontic treatment [10], we utilized the cutoff score of 4 or higher to define screen-positive mouth breathing. Consequently, participants were classified into a screen-positive group (HMB ≥ 4) and a screen-negative group (HMB ≤ 3).

#### 2.4.2. Cephalometric Analysis

**Figure 2:**
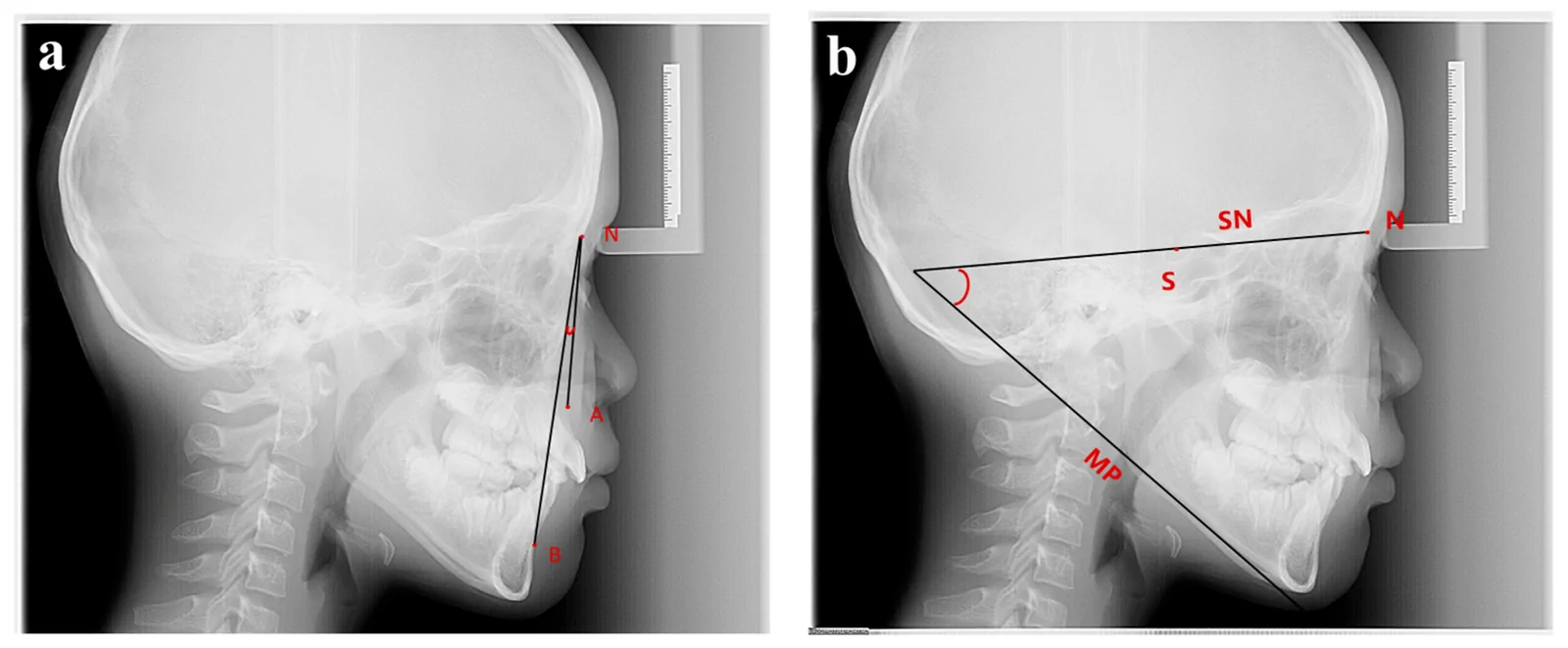
Schematic diagram of craniofacial angle measurements. (**a**) ANB angle. (**b**) SN-MP angle.

A lateral cephalometric radiograph was obtained for each participant and imported into Dolphin software (Version 11.95 Premium, Dolphin Imaging & Management Solutions, Chatsworth, CA, USA) for cephalometric analysis. The analysis was performed to evaluate sagittal and vertical skeletal patterns. Craniofacial measurements included: (1) The ANB angle, which is formed by the intersection of the lines connecting Point A (subspinale), Point N (nasion), and Point B (supramentale) (Figure 2a). This angle was used to evaluate the sagittal relationship between the maxilla and mandible. (2) The SN-MP angle, which represents the angle between the mandibular plane and the anterior cranial base plane (Figure 2b). This measurement was used to assess mandibular inclination and vertical skeletal pattern. Although the potential relevance of incisor position and inclination to lip posture was considered at the study design stage, the cephalometric analysis was prespecified to include two skeletal parameters, ANB and SN-MP. Therefore, dental parameters, including incisor position and inclination, were not included in the present analysis.

#### 2.4.3. Classification Criteria for Skeletal Patterns

Sagittal skeletal types were classified according to the ANB angle [17,18]: (1) skeletal Class I pattern, defined as 0° ≤ ANB ≤ 4°; (2) skeletal Class II pattern, defined as ANB > 4°; and (3) skeletal Class III pattern, defined as ANB < 0°. Vertical skeletal patterns were classified according to the SN-MP angle [17,19]: (1) low-angle pattern, defined as SN-MP < 27°; (2) normal-angle pattern, defined as 27° ≤ SN-MP ≤ 37°; and (3) high-angle pattern, defined as SN-MP > 37°.

#### 2.4.4. Measurement of LCS

**Figure 3:**
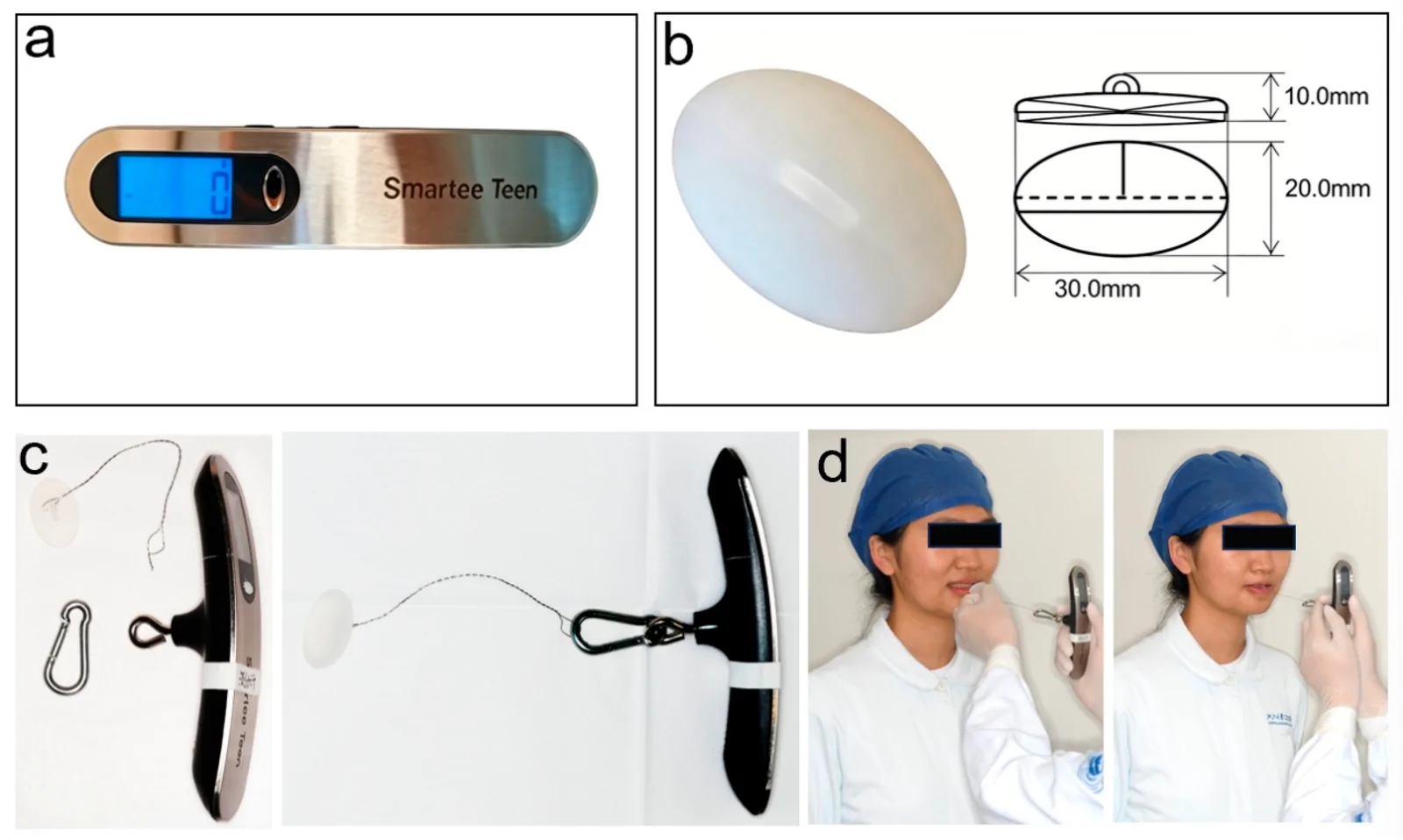
Apparatus and schematic for measuring lip-closing strength (LCS). (**a**) Digital dynamometer; (**b**) lip button; (**c**) complete measurement apparatus; (**d**) schematic illustration of the measurement procedure: The lip button is positioned between the upper and lower lips in front of the anterior teeth. Participants are instructed to maintain maximal lip closure while the dynamometer applies horizontal traction to the lip button.

LCS was quantified using a custom-designed lip-button measurement apparatus (Figure 3), which incorporated a digital force gauge (Smartee Teen digital force gauge, Smartee, Shanghai, China) to record the traction force generated during lip closure. The force gauge had a resolution of 0.01 N, with 1 g equivalent to 0.0098 N. All measurements were recorded directly in newtons and used for statistical analyses.

During measurement, participants were seated with their backs against a wall and their heads maintained in a natural position, ensuring that the orbital–auricular plane remained parallel to the floor. A lip button was placed between the upper and lower lips and positioned anterior to the incisors. Participants were instructed to exert maximal lip-closing force around the lip button while the examiner manually applied horizontal traction in a gradual and continuous manner until the lip button was released. Because traction was manually applied and the apparatus did not incorporate a motorized traction system, a fixed numerical traction rate was not prescribed, mechanically controlled, or recorded. The peak force immediately before the lip button was released was recorded.

Each measurement was repeated three times with a one-minute interval between trials. The mean value of the three measurements was calculated as the LCS value for each participant. A single trained examiner, blinded to participants’ skeletal classifications and screen-positive status for habitual mouth breathing, performed all measurements. Intrarater reliability was assessed in 30 randomly selected participants, yielding an intraclass correlation coefficient (ICC) of 0.93 (95% confidence interval: 0.88–0.96).

#### 2.4.5. Cephalometric Measurement and Reliability

All lateral cephalometric radiographs were analyzed by a single examiner who was blinded to the participants’ LCS values and HMB questionnaire results. Each radiograph was traced twice by the same examiner with a one-month interval between tracings, and the average value of the two tracings was used for subsequent statistical analyses. A second independent examiner repeated the measurements of 20 randomly selected radiographs to evaluate inter-examiner reliability.

Intra-examiner reliability was assessed by comparing the two measurements performed by the primary examiner. Inter-examiner reliability was assessed by comparing the measurements obtained by the primary and secondary examiners in the same set of 20 radiographs. Intraclass correlation coefficients (ICCs) were calculated using a two-way random-effects model with absolute agreement. The intra-examiner ICC for cephalometric parameters was 0.94 (95% CI: 0.88–0.97), and the inter-examiner ICC was 0.91 (95% CI: 0.83–0.95), indicating excellent measurement reliability.

### 2.5. Statistical Methods

Statistical analyses were performed using SPSS software (version 27.0; IBM Corp., Armonk, NY, USA). The normality of continuous variables was assessed using the Shapiro–Wilk test and Q-Q plots. Normally distributed data are presented as mean ± standard deviation (SD), while non-normally distributed data are presented as median with interquartile range (IQR). Categorical variables are expressed as frequencies and percentages. For statistical comparisons, age was categorized into yearly groups (7–12 years) based on chronological age.

For between-group comparisons, the independent-samples *t*-test was used for normally distributed continuous variables, and the Mann–Whitney U test was applied for non-normally distributed variables. Differences in LCS among the six age groups were assessed using one-way analysis of variance (ANOVA). Comparisons across three or more groups were performed using one-way analysis of variance (ANOVA), followed by Bonferroni-adjusted post hoc tests for pairwise comparisons. Effect sizes for ANOVA are reported as partial eta-squared (*η*^2^), and for Mann–Whitney U tests as *r*, with all effect sizes presented alongside their 95% confidence intervals.

To identify independent factors associated with lip-closing strength, variables showing significant associations in univariable analyses, including sagittal skeletal pattern and mouth-breathing screening status, were entered into a multiple linear regression model. Lip-closing strength was considered the dependent variable. Unstandardized regression coefficients (B), standardized coefficients (*β*), standard errors, 95% confidence intervals, and variance inflation factors were reported. Model fit was evaluated using the coefficient of determination (*R*^2^) and adjusted *R*^2^.

Intrarater reliability for LCS measurements and intra- and inter-examiner reliability for cephalometric tracings were assessed using intraclass correlation coefficients (ICCs) based on a two-way random-effects model for absolute agreement. ICC values were interpreted as follows: <0.50, poor; 0.50–0.75, moderate; 0.75–0.90, good; and >0.90, excellent. All statistical tests were two-tailed, and a *p*-value < 0.05 was considered statistically significant. All analyses were based on complete-case data, as no missing values were present for the variables included in the regression model.

## 3. Results

### 3.1. Demographic and Clinical Characteristics of the Study Population

A total of 379 participants (194 boys, 51.2%; 185 girls, 48.8%) with a mean age of 8.96 ± 1.50 years were included in the analysis. The distribution of sagittal skeletal patterns was as follows: skeletal Class II pattern (*n* = 246, 64.9%), skeletal Class I pattern (*n* = 95, 25.1%), and skeletal Class III pattern (*n* = 38, 10.0%). Based on the HMB questionnaire cutoff score (≥4), 113 participants (29.8%) screened positive for habitual mouth breathing. Detailed baseline characteristics are presented in Table 1.

The data are expressed as mean ± standard deviation or as *n* (%). Mouth breathing: screen-positive (HMB ≥ 4). Sagittal classification: Class I (0–4°), II (>4°), III (<0°) [17,18]; vertical classification: low-angle (<27°), normal-angle (27–37°), high-angle (>37°) [17,19]. LCS refers to lip-closing strength.

### 3.2. Association of LCS with Demographic and Craniofacial Characteristics

**Figure 4:**
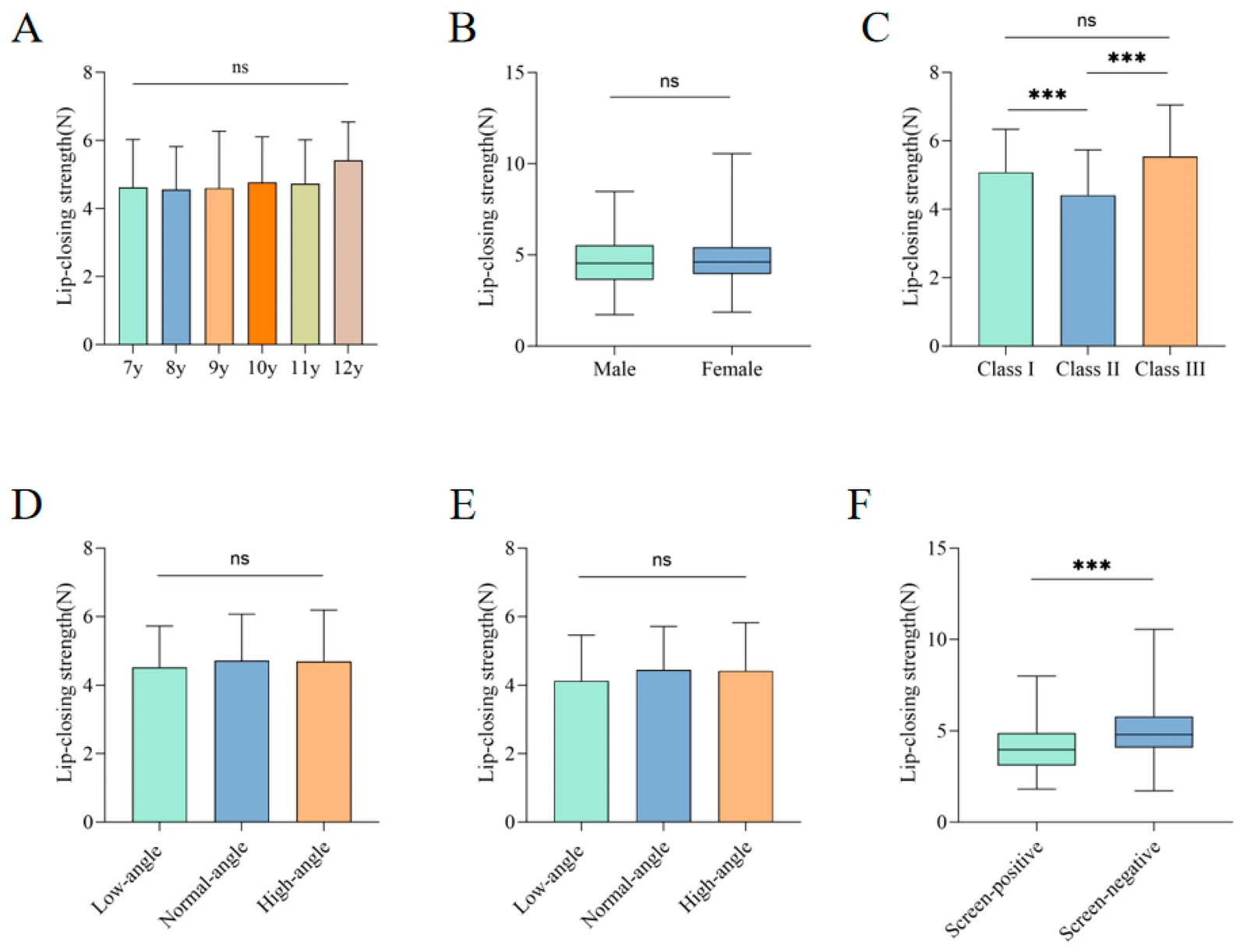
Comparison of lip-closing strength (LCS) according to demographic characteristics and skeletal/functional factors: (**A**) age, (**B**) sex, (**C**) sagittal skeletal patterns, where the Class II group exhibits significantly lower LCS than the Class I and Class III groups, (**D**) overall vertical skeletal patterns, (**E**) vertical patterns within the Class II subgroup (exploratory analysis), and (**F**) habitual mouth-breathing screening results based on the HMB questionnaire. Data are presented as mean ± standard deviation. Statistical significance is indicated as follows: ns, *p* ≥ 0.05; ***, *p* < 0.001.

No significant differences in LCS were observed according to age, sex, or vertical skeletal pattern (Table 2). In contrast, LCS differed significantly according to sagittal skeletal pattern and HMB questionnaire-based habitual mouth-breathing screening results. Specifically, participants with a skeletal Class II pattern showed lower LCS values than those with a Class I pattern (approximately 0.7 N lower) and a Class III pattern (more than 1.1 N lower). Participants who screened positive for habitual mouth breathing also showed lower LCS values than those who screened negative (approximately 0.83 N lower). The detailed results are presented in Table 2 and illustrated in Figure 4.

#### 3.2.1. Age and Sex

No significant differences in LCS were observed among different age groups (*F* = 1.585, *p* = 0.163, partial *η*^2^ = 0.021, 95% CI: 0.000–0.045). Mean LCS values were similar across age groups, ranging from 4.56 ± 1.26 N to 5.42 ± 1.12 N. Sex-related differences in LCS were also not observed. The median LCS was 4.54 N (IQR: 3.63–5.54 N) in males and 4.62 N (IQR: 3.95–5.42 N) in females (*Z* = −0.674, *p* = 0.500). Overall, LCS showed no significant differences according to age or sex (Figure 4A,B).

#### 3.2.2. Sagittal Skeletal Pattern

LCS differed significantly among sagittal skeletal patterns (Class I: 5.08 ± 1.26 N; Class II: 4.41 ± 1.33 N; Class III: 5.54 ± 1.51 N; *F* = 17.443, *p* < 0.001, partial *η*^2^ = 0.085, 95% CI: 0.037–0.140). Post hoc analysis showed that the Class II group had lower LCS values than the Class I group (mean difference: −0.67 N, 95% CI: −0.76 to −0.58) and the Class III group (mean difference: −1.13 N, 95% CI: −1.47 to −0.81). No significant difference was observed between the Class I and Class III groups (*p* = 0.069). These findings indicate that LCS differed according to sagittal skeletal pattern, with lower values observed in children with skeletal Class II pattern (Figure 4C).

#### 3.2.3. Vertical Skeletal Pattern

No significant differences in LCS were observed among different vertical skeletal patterns (low-angle: 4.52 ± 1.20 N; normal-angle: 4.72 ± 1.35 N; high-angle: 4.69 ± 1.50 N; F = 0.394, *p* = 0.675, partial *η*^2^ = 0.002). Within the skeletal Class II subgroup, LCS also did not differ significantly among vertical subtypes (low-angle: 4.13 ± 1.30 N; normal-angle: 4.45 ± 1.32 N; high-angle: 4.39 ± 1.37 N; *p* = 0.572). These subgroup analyses were exploratory (Figure 4D,E).

#### 3.2.4. Mouth-Breathing Screening Status

Children with a positive mouth-breathing screening result (HMB ≥ 4) showed significantly lower LCS values than those with negative screening results (median: 3.97 N, IQR: 3.10–4.89 N vs. 4.80 N, IQR: 4.09–5.79 N; median difference: 0.83 N; Z = −6.262, *p* < 0.001; Figure 4F).

### 3.3. Multivariable Analysis of Factors Associated with LCS

Multivariable linear regression analysis showed that, after adjustment for the included variables, skeletal Class I and Class III patterns were positively associated with LCS compared with skeletal Class II (Class I: B = 0.575, β = 0.180, *p* < 0.001; Class III: B = 0.867, β = 0.188, *p* < 0.001). In contrast, a positive mouth-breathing screening result was negatively associated with LCS (B = −0.808, β = −0.267, *p* < 0.001) (Table 3).

The regression model explained 15.2% of the variance in LCS (*R*^2^ = 0.152, adjusted *R*^2^ = 0.146; F (3, 375) = 22.457, *p* < 0.001). All VIF values were below 1.1, indicating no evidence of significant multicollinearity.

## 4. Discussion

### 4.1. Factors Associated with Lip-Closing Strength

Craniofacial growth and development are influenced by both genetic and environmental factors, and perioral muscle function contributes to the functional environment surrounding the dentofacial structures [20,21]. According to the classic “equilibrium theory”, the positioning of teeth within the alveolar bone exists within a functional environment shaped by the balance of forces exerted by the lips, tongue, and cheeks [20]. Changes in this functional balance may affect dental arch morphology and jaw development, and altered perioral muscle activity and breathing patterns have been suggested as potential contributors [20].

Although increasing attention has been given to the relationships among mouth breathing, perioral muscle function, and malocclusion, evidence regarding lip-closing strength (LCS) remains limited, especially in children. Previous studies have been restricted by small sample sizes or variations in assessment methods [22]. In this cross-sectional study involving 379 children aged 7–12 years, we found that LCS values were lower among children with skeletal Class II patterns and those with positive mouth-breathing screening results. After adjustment in multivariable analysis, sagittal skeletal pattern and mouth-breathing screening status remained significantly associated with LCS, whereas age, sex, and vertical skeletal pattern were not associated with LCS. These findings support the view that perioral muscle function is related to craniofacial characteristics and mouth-breathing screening status [22,23]. However, LCS should be considered as an objective functional assessment measure that provides additional information, rather than as a direct indicator of skeletal pattern or malocclusion.

### 4.2. Lip-Closing Strength, Age, and Sex

No significant association was found between LCS and chronological age within the 7–12-year range. Although this developmental period involves mixed dentition transition, tooth replacement, occlusal adaptation, and changes in oral function, perioral muscle development does not appear to increase continuously during school age. Consistent with previous evidence showing that lip pressure increases during early childhood but tends to stabilize after this period [3], our findings suggest that LCS remains relatively stable from 7 to 12 years of age. Fukami et al. [11] similarly reported substantial inter-individual variability in LCS during childhood, with limited evidence of a consistent age-related pattern. Moreover, Nogami et al. [24] demonstrated that LCS can be improved by short-term myofunctional training, and that the response may vary according to malocclusion and oral habits. Kugimiya et al. [25] further showed that LCS varies among healthy individuals and is associated with tongue pressure and masticatory performance, suggesting that LCS reflects broader aspects of oral function rather than force generation alone. Together, these findings indicate that LCS represents the current functional condition of the perioral musculature and may be influenced by functional factors beyond chronological age.

Sex differences were also not significant in our cohort, consistent with previous studies reporting no sex-related differences in LCS among school-aged children [15,22]. Fukami et al. [11] further suggested that sex-related differences become more apparent during adulthood rather than early childhood. This observation is supported by Barlow and Rath [12], who reported higher maximal LCS values in adult men, possibly due to differences in muscle development and neuromuscular function after puberty. Taken together, the absence of a sex difference in our cohort contrasts with adult-based studies reporting higher LCS values in men [12], and is consistent with the concept that sex-related differences in perioral muscle function may become more apparent after puberty.

### 4.3. Lip-Closing Strength and Sagittal Skeletal Pattern

The sagittal skeletal pattern showed the strongest association with LCS among the evaluated craniofacial characteristics. Children with skeletal Class II exhibited significantly lower LCS than those with Class I or Class III patterns, and this association remained significant after multivariable adjustment. This finding may be explained by the altered soft-tissue environment associated with Class II skeletal relationships. Mandibular retrusion and maxillary incisor protrusion may increase the functional demand required to achieve lip closure, potentially requiring greater activity of the perioral muscles [1].

Previous studies have suggested that abnormal perioral muscle function may be associated with sagittal skeletal characteristics. Saker et al. [26] reported that altered perioral muscle activity was associated with increased ANB angles, supporting the relationship between craniofacial morphology and oral muscle function. Our finding that skeletal Class II was independently associated with reduced LCS is therefore consistent with and extends the previous observations of Jung et al. [1] and Saker et al. [26] by examining this association using skeletal classification together with an objective measure of lip function. However, the direction of this relationship remains unclear. A bidirectional relationship between jaw position and lip function has been proposed, although causality cannot be determined from the present cross-sectional study. Skeletal morphology may influence perioral muscle function by altering lip posture and the functional conditions required for oral closure. Conversely, changes in lip pressure may contribute to dental compensation by modifying the balance of forces acting on the anterior teeth.

Interestingly, LCS did not differ significantly between skeletal Class III and Class I children. Unlike Class II patterns, the anterior positioning of the mandible in Class III may facilitate lip closure, although this hypothesis requires further investigation. This finding is partially consistent with Kurabeishi et al. [13], who reported an association between skeletal classification and orofacial muscle pressure in Japanese children, although their study focused on tongue pressure rather than lip function. Together, these findings suggest that reduced LCS is particularly associated with skeletal Class II patterns and may provide additional functional information during the evaluation of sagittal skeletal characteristics in mixed-dentition children.

### 4.4. Lip-Closing Strength and Vertical Skeletal Pattern

Evidence regarding the relationship between lip strength and vertical facial pattern remains limited. In this cohort, LCS did not differ among low-, normal-, and high-angle groups, nor among vertical subtypes within the skeletal Class II subgroup. These findings differ from previous clinical descriptions suggesting that some high-angle Class II patients may present with lip incompetence, anterior open-bite tendency, and increased mentalis activity. However, the absence of an association between vertical pattern and LCS does not exclude a potential relationship between vertical facial morphology and perioral function. Vertical skeletal development is influenced by multiple factors, including genetic background, mandibular growth direction, airway conditions, and occlusal forces [27,28]. Therefore, LCS alone may not fully reflect the complex mechanisms involved in vertical facial growth patterns.

Although Jung et al. [1] identified an association between vertical characteristics and lip strength in adult Class II patients, their findings were based on a mature skeletal population. In contrast, children aged 7–12 years are still undergoing active craniofacial growth and mandibular remodeling, and vertical facial proportions may not yet be fully established. This ongoing developmental process may partially explain the limited association between vertical skeletal pattern and LCS observed in our cohort. The absence of a significant association in children, together with the findings reported in adults, suggests that the relationship between vertical skeletal characteristics and lip function may vary across developmental stages. Clinically, these findings suggest that LCS should not be used alone to distinguish high-angle and low-angle malocclusions during the mixed dentition stage. Instead, LCS may provide complementary functional information, while vertical skeletal evaluation should continue to rely on conventional cephalometric assessment.

### 4.5. Lip-Closing Strength and Mouth-Breathing Screening Status

This study demonstrated that children in the screen-positive group (HMB ≥ 4) exhibited significantly reduced LCS. This finding is consistent with the findings of Masutomi et al. [29], who reported that lip-closing force, tongue pressure, and masticatory efficiency were lower in adolescents with mouth-breathing habits than in nasal breathers. This is also in line with previous studies linking habitual mouth breathing to craniofacial adaptations, including maxillary arch constriction and a high-arched palate [5,30].

The observed association may be related to functional changes reported in children with mouth-breathing habits. These changes may include reduced activation of the orbicularis oris during resting oral posture, together with a lowered tongue position and increased inward pressure from the buccinator muscles on the maxillary arch [31]. Masutomi et al. [29] further showed that these functional differences involve not only lip function but also tongue pressure and masticatory efficiency, suggesting that mouth-breathing-related functional changes may involve multiple aspects of oral function rather than the lips alone. These alterations may disturb the balance of intraoral and extraoral forces and may be associated with reduced LCS. A reciprocal relationship is also possible: prolonged mouth breathing may be associated with reduced perioral muscle activity, while lower lip strength may make effective lip seal more difficult and potentially affect nasal breathing stability. Beyond functional changes, mouth breathing has been discussed as part of a broader condition involving factors such as adenotonsillar hypertrophy and altered craniofacial growth patterns, which may further influence perioral function over time [32]. In our study, multivariable regression showed that mouth-breathing screening status remained independently associated with lower LCS after adjustment for sagittal skeletal pattern (B = −0.808, 95% CI: −1.099 to −0.517).

### 4.6. Clinical Implications

These findings suggest that LCS may provide adjunctive functional information rather than serve as a standalone diagnostic or treatment-planning tool. Although significant differences were observed by sagittal skeletal pattern and mouth-breathing screening status, the between-group differences were modest (0.575–0.867 N and 0.808 N, respectively), corresponding to approximately 0.36–0.55 SD of LCS variability (SD = 1.58 N). Their clinical significance remains uncertain, given the absence of an established LCS threshold and treatment-outcome data in this cross-sectional study. Emerging evidence indicates that LCS may be modified through functional and orthodontic interventions: lip and facial muscle training has been reported to improve LCS in children [33], and orthodontic treatment with premolar extractions has also been associated with increased maximum lip-closing force [34]. These findings suggest that LCS may have potential relevance to functional assessment, although its role in guiding treatment planning remains to be established.

In clinical practice, a reduced LCS value may provide additional functional information and may encourage clinicians to evaluate lip seal and breathing patterns alongside conventional skeletal measurements, particularly in children undergoing orthodontic assessment. When evaluating mixed-dentition patients, clinicians may consider not only conventional skeletal and dental parameters but also functional parameters including LCS and breathing patterns.

For children with skeletal Class II patterns or positive mouth-breathing screening results, comprehensive orthodontic evaluation may benefit from considering both morphological characteristics and perioral functional status. Further longitudinal studies are needed to determine whether LCS has prognostic value, whether a clinically meaningful threshold can be established, and whether changes in LCS following functional or orthodontic interventions are associated with clinically meaningful treatment outcomes.

### 4.7. Limitations

Several limitations should be considered when interpreting our findings. First, the cohort was recruited from a single pediatric dentistry center and included a relatively high proportion of children with skeletal Class II (64.9%) and positive mouth-breathing screening results (29.8%). Because children seeking orthodontic assessment may differ from community-based pediatric populations, and skeletal Class II has been reported to show associations with sleep-disordered breathing-related characteristics [10], the generalizability of our findings should be interpreted with caution.

Second, mouth-breathing status was assessed using a parent-reported questionnaire without objective airway confirmation. Although the HMB questionnaire has been validated [16], questionnaire-based screening cannot replace clinical assessment of mouth-breathing status. Moreover, mouth breathing in children may coexist with nasal obstruction and sleep-disordered breathing [35]. Current consensus recommendations emphasize that clinical evaluation of resting lip posture and tongue position remains important for accurate assessment of mouth-breathing patterns [36]. Future studies should incorporate objective evaluations, including clinical observation of resting lip posture, airway examination, or other validated respiratory assessments.

Third, the study was primarily designed to evaluate differences in LCS between skeletal Class II and non-Class II children, and the sample size calculation was based on this primary comparison. Consequently, analyses involving smaller subgroups—such as skeletal Class III children (*n* = 38), the 12-year age group (*n* = 23), or vertical subtypes within the Class II subgroup—had limited statistical power and should therefore be interpreted as exploratory rather than confirmatory.

Finally, although LCS measurement using the custom device showed excellent intrarater reliability (ICC = 0.93, 95% CI: 0.88–0.96), traction was applied manually, and its velocity was neither mechanically controlled nor recorded. This may have introduced some examiner-dependent variation in traction rate. In addition, the device has not yet been cross-validated against established reference instruments, which may limit direct comparisons with studies using other measurement systems. Future studies using a motorized or rate-controlled traction system together with standardized and validated measurement devices may further improve the standardization, comparability, and reproducibility of LCS measurements across populations and research settings.

In addition, the position and inclination of the incisors may also affect lip posture and therefore represent potential confounding factors in the association with LCS [37]. Because these dental variables were not prespecified in the cephalometric protocol, their contribution could not be evaluated separately from skeletal morphology in the present study. Future studies incorporating relevant dental cephalometric parameters, including measures of incisor position and inclination [38], may help distinguish the effects of skeletal morphology from dental compensation on lip closure. Other potentially relevant demographic and clinical factors were not systematically assessed in the present study; therefore, residual confounding from unmeasured factors cannot be excluded.

## 5. Conclusions

In this cross-sectional study of 379 children aged 7–12 years undergoing orthodontic assessment, children with a skeletal Class II pattern and those who screened positive for mouth breathing had lower LCS. In the adjusted analysis, LCS was 0.575 N higher in children with Class I and 0.867 N higher in those with Class III than in those with Class II, while a positive mouth-breathing screening result was associated with a 0.808 N lower LCS. These differences were modest, and their clinical significance remains uncertain because no established clinically meaningful threshold for LCS is currently available. Thus, the observed group-level associations do not establish LCS as a standalone diagnostic or treatment-planning indicator. Future multicentre studies incorporating objective airway evaluations and longitudinal follow-up are needed to determine the clinical and prognostic relevance of LCS.

## Figures and Tables

**Table 2:** Lip-closing strength (LCS) across participant subgroups.

Variable	Category	*n*	LCS (N) Mean ± SD	Statistic	*p*-Value	Effect Size (95% CI)
Age group (years)	7	70	4.62 ± 1.41	*F* = 1.585	0.163	partial *η*^2^ = 0.021 (0.000, 0.045)
	8	104	4.56 ± 1.26			
	9	72	4.60 ± 1.67			
	10	59	4.77 ± 1.34			
	11	51	4.73 ± 1.29			
	12	23	5.42 ± 1.12			
Sex	Male	194	4.54 (3.63, 5.54)	*Z* = −0.674	0.500	*r* = 0.068 (−0.067, 0.135)
	Female	185	4.62 (3.95, 5.42)			
Sagittal skeletal pattern	Class I	95	5.08 ± 1.26	*F* = 17.443	<0.001	partial *η*^2^ = 0.085 (0.037, 0.140)
	Class II	246	4.41 ± 1.33			
	Class III	38	5.54 ± 1.51			
Vertical skeletal pattern	Low-angle type	44	4.52 ± 1.20	*F* = 0.394	0.675	partial *η*^2^ = 0.002 (0.000, 0.016)
	Normal-angle type	189	4.72 ± 1.35			
	High-angle type	146	4.69 ± 1.50			
Mouth breathing	Screen-positive	113	3.97 (3.10, 4.89)	*Z* = −6.262	<0.001	*r* = 0.322 (0.228, 0.409)
	Screen-negative	266	4.80 (4.09, 5.79)			

Note: LCS: lip-closing strength; SD: standard deviation; CI: confidence interval. Effect sizes: partial *η*^2^ for ANOVA; *r* for Mann–Whitney U tests.

**Table 3:** Multiple linear regression analysis of factors associated with lip-closing strength (N).

Variable	B (Unstandardized)	SE	*β* (Standardized)	*t*	*p*-Value	95% CI for B	VIF
Constant	4.698	0.098	-	48.056	<0.001	4.506–4.890	-
Skeletal facial pattern (reference: Class II)							
Class I	0.575	0.156	0.180	3.687	<0.001	0.268–0.882	1.052
Class III	0.867	0.229	0.188	3.784	<0.001	0.416–1.317	1.090
Mouth-breathing habit (reference: Screen-negative)							
Screen-positive	−0.808	0.148	−0.267	−5.460	<0.001	−1.099–−0.517	1.055

Note: Dependent variable: lip-closing strength (N). VIF = variance inflation factor; all VIF values < 10, indicating no significant multicollinearity. Model summary: *R* = 0.390, *R*^2^ = 0.152, adjusted *R*^2^ = 0.146; *F* (3, 375) = 22.457, *p* < 0.001. All VIF values < 10, indicating no significant multicollinearity.

## Data Availability

The data presented in this study are available upon request from the corresponding author due to privacy and ethical restrictions.
